# Physical effort and task errors influence the choice for cognitive offloading

**DOI:** 10.1007/s00426-025-02186-1

**Published:** 2025-10-15

**Authors:** Rouven Aust, Patrick P. Weis, Wilfried Kunde

**Affiliations:** Department of Psychology, University of Würzburg, Röntgenring 11, Würzburg, 97070 Germany

## Abstract

**Abstract:**

Minimizing effort is a principle widely accepted to govern human behavior. We revisited this principle in an extended rotation paradigm. Participants freely chose to solve an object comparison task by either mental or manual rotation of one of two simultaneously presented objects. We manipulated the required force of manual rotation, stimulus complexity, as well as the angular mismatch between both objects, while carefully eliminating confounds of physical effort with time. Our study revealed that both physical and mental effort affect strategy choice. Additionally, strategy choice in a certain trial was influenced by error commissions in the previous trial. These error-induced strategy switches were asymmetric: participants were more inclined to switch following mental than manual rotation errors. Our results suggest that human reactions to error commission when using external tools may differ in a qualitative manner from reactions to error commission during mental endeavors. Theoretical implications are discussed especially in the context of the entanglement of physical effort and time.

**Public Significance Statement:**

Humans possess the ability to solve problems using either mainly mental or physical resources. For example, objects can be rotated in the mind or physically. Here, we show that when comparing the effort associated with both means, they are both considered for decision making. Furthermore, making a mistake seems to evoke different reactions depending on the means with which the error was made. Considering such different costs and benefits is vital for both designing and working in tech-infused environments that support problem solving.

**Supplementary Information:**

The online version contains supplementary material available at 10.1007/s00426-025-02186-1.

## Introduction

Humans possess the remarkable faculty to simulate actions mentally instead of having to execute them bodily. When moving out, some people might mentally rotate a heavy sofa to figure out how it best fits through the staircase instead of physically trying. However, other people might directly lift up the sofa without initial mental rotation. One major aspect that connects both options is that both come with the exertion of effort. Effort can be described as *“subjective intensification of mental and/or physical activity in the service of meeting some goal.”* (Inzlicht et al., 2018, p. 338). This is a broad and debatable conception (Massin, 2017). But for the present purpose it suffices to say that effort can be manipulated. Increases of mental activity, in terms of difficulty or a cognitive task arguably increases mental effort, whereas increases of physical activity in terms of muscles forces increases physical effort. Here, our main research question is how cognitive and physical effort shape our strategy choices during problem solving.

It is widely accepted that humans frequently avoid effort (e.g. Kahnemann, 1973; Fiske & Taylor, 1991). Already the french philosopher Pierre-Louis Moreau de Maupertuis has proposed that all behavior – be it human or otherwise – strives to minimize some form of action. In other words, people often choose to behave in a way that allows to reach goals with the least effort needed to do so (*principle of least action*, Maupertuis, 1750; see also *law of less work*, Hull, 1943). Empirical work shows that effort is not only relevant when choosing between mainly physically demanding tasks (Cheval, 2021; De Camp, 1920; Lieberman, 2015; Morsella et al., 2011; Pontzer et al., 2015) but also when choosing between mainly cognitively demanding tasks (Botvinick & Rosen, 2009; Kool et al., 2010; Kool & Botvinivk, 2018; Neszmélyi & Pfister, 2024; Shenhav et al., 2017). According to the opportunity cost model, effort can be construed as the experienced cost of choosing a cognitive task relative to other available options (Kurzban, 2013).

However, until recently, most of this past research had looked at either cognitive or physical effort in isolation and not in combination. Bridging this gap has been an avenue of recent work to test whether the principle of least action also stands when one of two tasks has to be selected, of which one entails mostly cognitive and the other mostly physical effort. Related empirical studies measured how much effort individuals were willing to exert in one domain to reduce the exertion of effort in the other domain (Anderson & Lee, 2023; Chiu & Gilbert, 2024; Feghhi & Rosenbaum, 2019; Janczyk et al., 2022; Potts et al., 2018; Rosenbaum, 2014, 2019; Vogel et al., 2020). When focusing on reducing the costs of mental task completion, the choice to solve a task physically is often denoted as cognitive offloading (Risko & Gilbert, 2016). This can include the use of a (digital) tool like when flipping a PDF-page by 90° to better read the caption on the y-axis of a plot) or to use the own body like when tilting one’s head to do the same. Results consistently suggest that human agents *can* trade-off physical and mental effort when choosing between offloading a task and mental task completion. So why having another look at the trade-off between mental and physical effort?

First, in many of these studies participants were explicitly instructed to “do what’s easier”, that is choose the task alternative, that participants thought will be less effortful (Feghhi & Rosenbaum, 2019; Janczyk et al., 2022; Potts et al., 2018; Rosenbaum, 2014, 2019). Even though the principle of effort minimization would predict that people do exactly that, the instruction may counterintuitively undermine the free choice character of the task and introduce a choice bias. This is due to the fact that effort is not necessarily and not always an undesirable state to be avoided whenever possible but can sometimes increase the value of an associated action (Inzlicht et al., 2018; Norton et al., 2012). In other words, there seem to be exceptions to the principle of least effort. Many species, including humans, do sometimes choose to invest effort to reach a certain goal, even if this goal could be achieved with less effort exertion (Inglis et al., 1997; Wu et al., 2022). Moreover, in many societies the effort one exerts can become a secondary reinforcer (Eisenberger, 1992) and thus may not be avoided contrary to what the principle of least effort would predict. All in all, while there is convincing evidence in favor of effort minimization, there is also evidence suggesting effort-seeking behavior. We thus deem it pressing to look at choices between mentally and physically effortful strategies without instructions targeted at reducing effort.

Second, we hold that more care needs to be taken to disentangle the influence of effort and time on strategy choice. While it is true that in real-life settings more effort often goes hand in hand with requiring more time, we deem it imperative to separate both factors in experimental settings, especially because time is known to have a robust influence on strategy choice (e.g.; Gray et al., 2006; Gray & Fu, 2004; Kurzban, 2013; Mendl & Dreisbach, 2024; Potts et al., 2018). However, most studies on effort minimization conflate physical effort and time. For example, although multiple compared to single mouse clicks (Chiu & Gilbert, 2024) and slow compared to fast computer mice (Morsella et al., 2011) may require more effort, they inherently also require more time. Physical effort had even been equated with task duration (Davey, 1973), which is conceptually highly problematic. The question therefore arises whether task choices in experiments not controlling for time are attributable to differences in effort or possibly to differences in task durations. Hence, here, we focused on means to reduce the time-effort confound as far as possible.

Third—we conceived this possibility *a posteriori*—failures like providing a wrong answer in one trial are known to trigger adaptive responses in following trials, which might differ depending on whether physical or mental effort had been exerted. Effort is intricately linked to success and thus investing effort would only make sense if it ultimately resulted in adequate performance. With a high probability of failure (e.g. errors), even investing little effort appears pointless. Accordingly, recent experience of failure with a certain option shapes subsequent choices of that option. Specifically, if participants can choose among two unrelated tasks, for example between judging either the motion or color of an object, they tend to switch to the respectively other task after committing an error in the just chosen task (e.g. Spitzer et al., 2022). One may thus assume that the same happens when people choose between two available strategies for solving the same task rather than choosing between two different tasks. Yet, one notable difference between task choices and strategy choices, is that the task goal (e.g. judging motion or color) fundamentally differs for the former but remains the same for the latter (e.g. reaching the same goal by either one or the other strategy). So while error-induced task switches could stem from shying away from a goal that had not been successfully reached, the goal when choosing between a more physical and a more mental way to solve a task stays the same. While these analyses were not pre-registered, we deemed it worth exploring whether the same switch tendency after errors that had been reported for voluntary task switching (Embrey et al., 2025; Spitzer et al., 2024) can be observed when choosing between physical and mental means to solve a task.

In a nutshell, in the following two experiments, we asked participants to make a probably ancient choice, namely to rotate an object either manually or mentally (Weis & Wiese, 2019; Weis & Kunde, 2024; Wohlschläger & Wohlschläger, 1998). We studied how the cognitive costs of mental rotation and the physical costs of manual rotation shape strategy choice. Additionally, we explored how errors after both manual and mental rotation shape subsequent strategy choices.

## Experiment 1

**Fig. 1:**
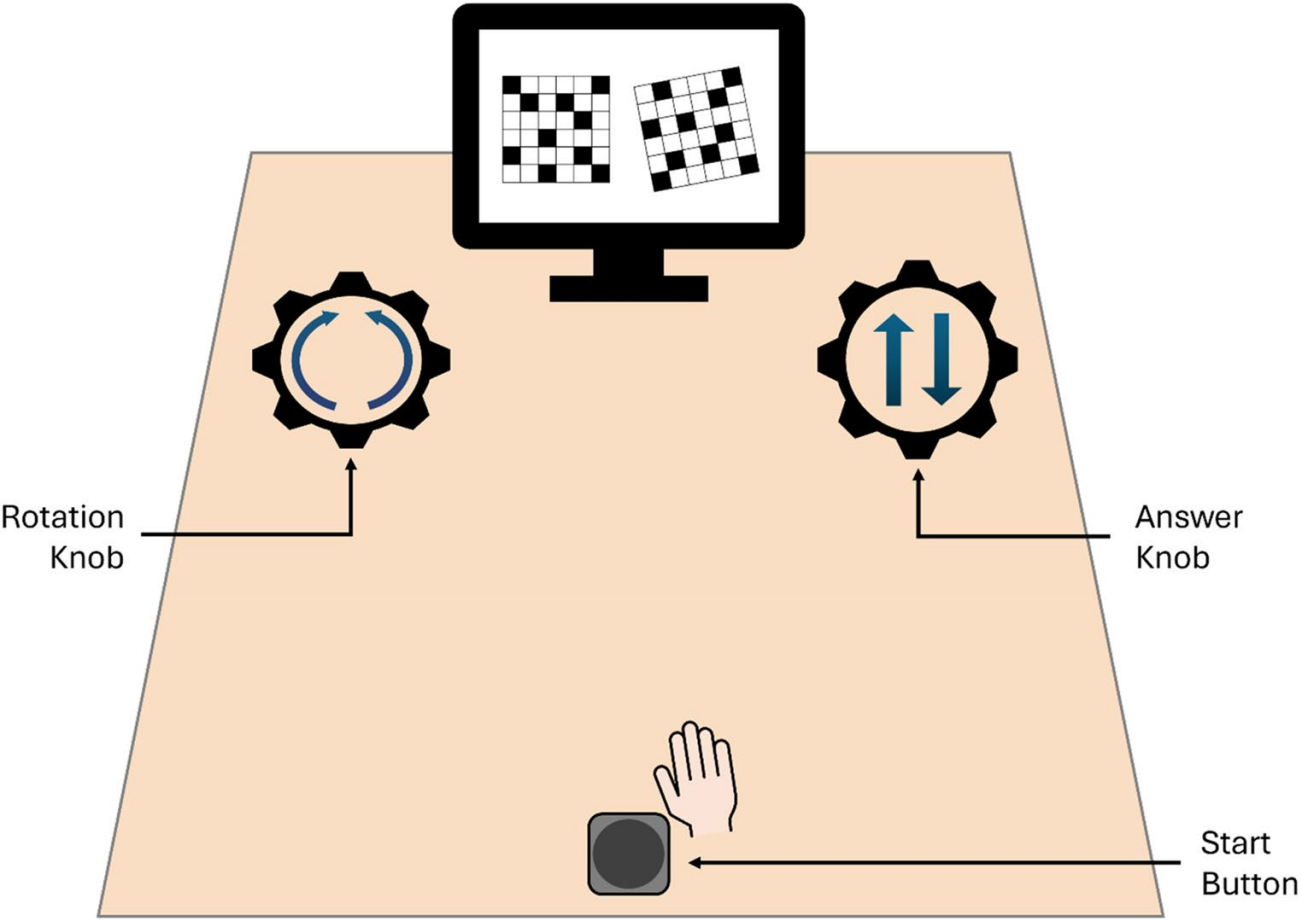
Experimental setup in Experiments 1 and 2

Participants were to judge whether two visual objects presented in varying orientations were either identical or mirror-reversed. The task could be solved by mental object rotation alone (Shepard & Metzler, 1971). Alternatively, mental rotation could be offloaded to manually turning one of the objects by a rotation knob (see Fig. 1)^1^ Participants were first acquainted to both means of task completion before giving them the opportunity to freely choose among them. We varied the physical costs of offloading by having a rotation knob which required either low or high forces to be turned. Moreover, we varied the costs of mental rotation by having stimuli of either low or high complexity, as previous research suggests that mental rotation speed slows down with increasing object complexity (Bethell-Fox & Shepard, 1988). We expected that mental effort would increase offloading rate, whereas physical effort would decrease it. Finally, we analyzed whether participants were inclined to offload the task after committing an error as compared to after correct task completion. Both experiments were preregistered on the Open Science Framework and can be accessed at: https://osf.io/hwb3p/.

## Methods

### Participants

We conducted an a priori power analysis using G*Power (Faul et al., 2007). Due to the fact that many earlier studies (Anderson & Lee, 2023; Chiu & Gilbert, 2024; Janczyk et al., 2022; Potts et al., 2018) found very large effect sizes of physical effort ($\:{\eta}_{\text{p}}^{2}=0.30-0.68$) and we have reason to believe that these studies systematically overestimate this effect, we based our power analysis on a pilot study which yielded a considerably smaller effect size ($\:{\eta}_{\text{p}}^{2}=0.14$). Since this pilot study was not able to capture physical effort but rather task duration^2^ in our power analysis we aimed at detecting a medium-sized effect ($\:{\eta}_{p}^{2}=0.10$) by means of a repeated measures ANOVA and linear-mixed models. This power analysis yielded a minimum required sample size of *N* = 36 participants with a power of 1-β = 0.8 and α = 0.05. We therefore collected a total of 36 analyzable data sets via an online participant pool management platform of the University of Würzburg. Both experiments were performed in accordance with the declaration of Helsinki (Rickham, 1964) and had been approved by the local ethics committee (Ethikkommission des Institutes für Psychologie der Humanwissenschaftlichen Fakultät der Julius-Maximilians-Universität Würzburg, GZ 2024-14). The study took about 75 min. All participants gave their written consent before participation and received financial compensation of 15€. We excluded any participants, for which technical failures of our apparatus (motion tracker)^3^ arose in collecting the data (*n* = 8, 14.8%), as well as those who did not meet our inclusion criteria (*n* = 6, 11.1%). Our final sample consisted of 36 participants (25 females, $\:{M}_{age}$ = 30 years, $\:{range}_{age}$ = 20–62 years).

### Apparatus

Participants sat in front of an LCD monitor (21-in, Acer G226HQL) with a resolution of 1920 × 1080 pixels and a 60-Hz refresh rate. Stimuli were presented using the PsychoPy software (version 2023.2.3, Peirce et al., 2019). On the table in front of the monitor there was a single keyboard button, which served as a starting point for all hand movements. Two custom made knobs were placed further back on the table with a distance of 50 cm between the knobs as well as between each knob and the start button to form an equilateral triangle. A schematic illustration of the experimental setup can be found in Fig. 1.

### Procedure and task

After giving consent to participate, participants read instructions concerning the task and starting procedure of each trial. Participants were seated at a distance of approximately 60 cm from the computer screen and completed 36 practice trials of the Extended Rotation Task before experimental trials started. In this task participants compare a base stimulus and a working stimulus, to indicate whether they are identical or mirror reversed. The right stimulus always being the working stimulus was rotated in one of six possible orientations (50°, 100°, 150°, 210°, 260° and 310°). While the 50° and 310° orientations are both 50° away from the canonical orientation (the same goes for 100° and 260° as well as 150° and 210°) using both orientations increases variability of the trial structure and therefore reduces familiarity with stimuli. Additionally, in half of all trials the working stimulus was presented in a mirror reversed manner (“non identical”) as opposed to the base stimulus on the left side of the screen. One of the knobs could be turned to afford manually rotating the working stimulus on the computer screen against a varying resistance. This resistance was set to about 0.5 Newton meters (Nm) for the low resistance condition and to about 1.1 Nm for the high resistance condition. Our rationale was that such a manipulation should not be confounded with time, since turning the knob in the high resistance condition should be doable just as fast as in the low resistance condition by simply investing more physical effort.

The answer knob was either pushed down or pulled up to indicate whether participants believed the working stimulus to be mirror-reversed or to match the base stimulus. This setup leads to two possible strategies to solve the task, one mental rotation strategy and one manual strategy for which the rotation knob is used. Participants were asked to only use one hand for all task requirements. Half the participants used the right hand, the other half the left hand, irrespective of what participants reported to be their dominant hand. The participants’ goal was to solve the task as quickly and as accurately as possible. A trial in the free choice condition counted as offloaded if the rotation knob had been turned. Response time was the time between stimulus presentation and entering the response (identical vs. mirror reversed). We would like to point out that offloading a trial required participants to move their hand twice the distance (first to the rotation knob and then to the answer knob) of what is required when mentally solving the task (only to the answer knob). This makes it difficult to compare the absolute levels of response times of both strategies. Interpreting response time differences between the two strategies should therefore only be done with extreme caution.

The experiment followed the choice/no-choice method (Siegler & Lemaire, 1997). After completing the practice trials, three Forced-Choice blocks of the task had to be worked through, one of which only allowed to use the mental strategy and two of which only allowed to use the offloading strategy (one for each level of knob resistance). In those blocks it was either impossible to indicate an answer before having manually turned the working stimulus into the 0°-orientation (Forced-Choice: Offloading) or impossible to use the rotation knob at all (Forced-Choice: Mental). All forced-choice blocks were performed at the beginning of the experiment, followed by two free-choice blocks, again one for each level of knob resistance. The order of the blocks within the forced-choice and within the free-choice blocks was randomized. We included Forced-Choice blocks to familiarize participants with the two strategy options, and as manipulation checks for our independent variables.

**Fig. 2:**
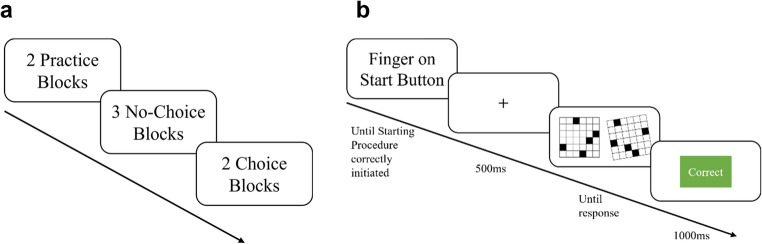
Experimental and Trial Structure of Experiment 1 and 2. **a**) Study design of Experiment (1) In Experiment 2, forced-choice blocks were removed. **b**) Trial Structure of Experiments 1 and 2. Pressing the start button for 500 ms led to presentation of stimuli. A response could be given without time limit

Before the free-choice blocks commenced, participants were told that they could now choose either strategy to solve the task. They were instructed to – on each trial – choose the strategy, which allows them to solve the task as fast and as accurately as possible. It was also stated that a combination of both strategies was possible, that is first rotating a stimulus manually up until a point when a mental continuation may be time or effort saving. Cases in which participants used this technique were still classified as offloaded trials. A schematic overview of the experimental and trial structure can be found in Fig. 2.

Before each trial the screen showed basic instructions on how to initiate the trial. This was done by placing the proximal phalanx of the middle finger of the according hand over the start button. While pressing the start button for 500ms a fixation cross was shown in the screen center, after which both stimuli were presented. There was no time limit for each trial so that a trial ended when the answer knob registered a key press.

### Stimuli

**Fig. 3:**
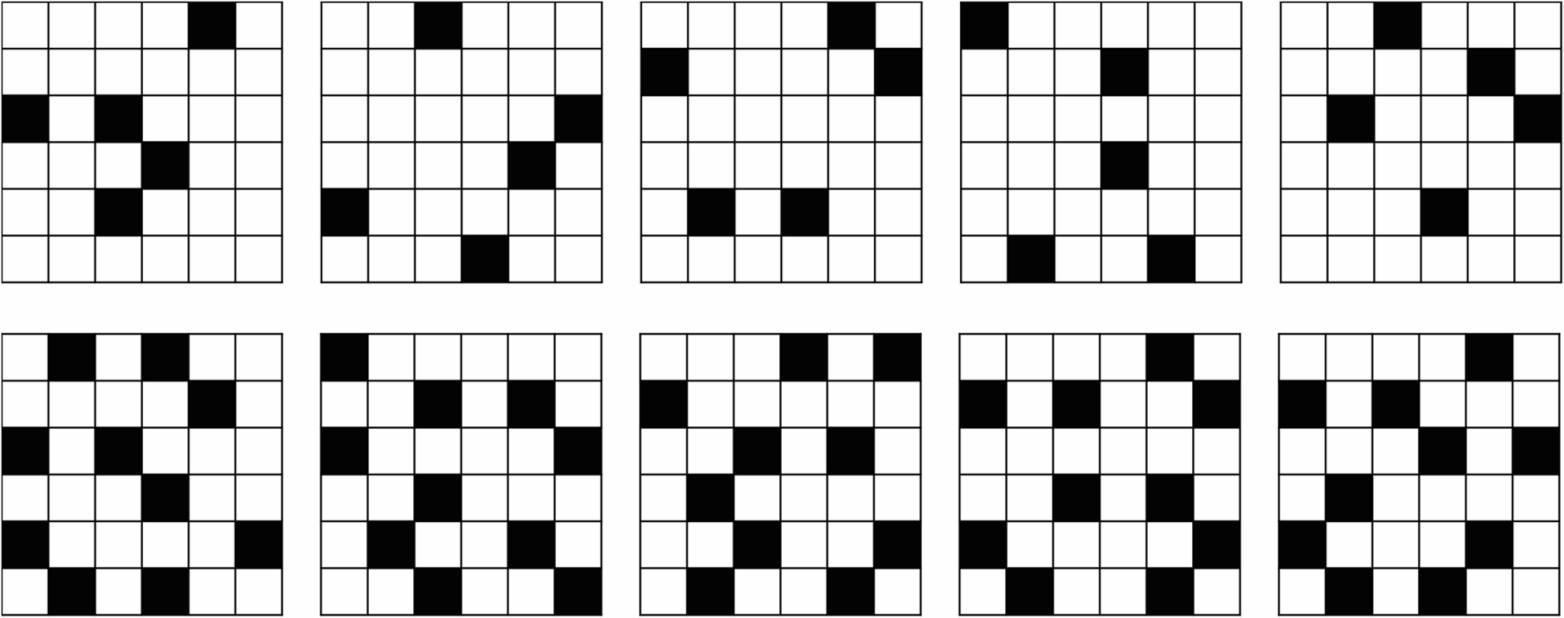
Subset of stimuli for Experiments 1 and 2. Top row depicts stimuli of low complexity. Bottom row depicts stimuli of high complexity

As stimuli 36 custom-designed 6 × 6 matrices with white background and black coloring were used, based on the stimuli used by Bethell-Fox and Shepard (1988). These stimuli were constructed to adhere to a list of two criteria to achieve a stimulus complexity effect. First, it was necessary to create a large number of different stimuli, in order to reduce any familiarity effects (Bethell, 1988; Folk, 1987). Second, no two colored fields were supposed to be directly adjacent because this is known to facilitate pattern recognition and therefore mental rotation (e.g. Shevelev et al., 2003). We aimed to construct two different levels of stimulus complexity. Therefore, 18 stimuli were made to have five colored fields (low stimulus complexity), while a second set of 18 stimuli was made to have ten colored fields (high stimulus complexity; see Fig. 3). Stimuli were constructed using Microsoft Power Point.

### Design

The experiment followed a 2 (Knob Resistance) x 2 (Stimulus Complexity) x 3 (Working Stimulus Orientation) repeated measures design, with block-wise manipulation of physical effort and trial-wise manipulation of stimulus complexity and working stimulus orientation. The position of each knob (offloading knob left vs. right), which hand was used to execute the task (left vs. right hand) and the mapping of the answer knob (up vs. down) to indicate matching stimuli was counterbalanced across participants. Dependent variables were offloading rate, response times and accuracy.

### Data analysis

All analyses were done using linear or logistic mixed-effects models accounting for the hierarchical data structure. For this purpose, the independent variables were input as fixed factors nested within participants for which a random intercept was modeled.

#### Forced choice blocks

For the forced choice blocks, response times were analyzed using a linear mixed model and errors using a logistic mixed model. As predictors, both models included the given strategy (mental vs. manual), the stimulus complexity (high vs. low) and the stimulus orientation (50°, 100°, 150° misorientation), as well as all possible interactions. This model did not include knob resistance as predictor, as knob resistance was only manipulated for the two manual forced choice blocks. We formalized this model as:

1 $$\begin{array}{c}Response\;Time=\beta_0+\beta_1X_1+\beta_2X_2+\beta_3X_3+\\\beta_{12}X_1X_2+\beta_{13}X_1X_3++\beta_{23}X_2X_3+\beta_{123}X_1X_2X_3\end{array}$$

where $\:{X}_{1}$ = Selected Strategy, $\:{X}_{2}$ = Stimulus Complexity and $\:{X}_{3}$ = Stimulus Orientation.

Therefore, a second linear mixed model was conducted only for the two manual forced choice blocks with the following predictors: knob resistance (high vs. low), stimulus complexity and stimulus orientation as well as all possible interactions.

2 $$\begin{array}{c}Response\;Time=\beta_0+\beta_1X_1+\beta_2X_2+\beta_3X_3+\\\beta_{12}X_1X_2+\beta_{13}X_1X_3++\beta_{23}X_2X_3+\beta_{123}X_1X_2X_3\end{array}$$

where $\:{X}_{1}$ = Knob Resistance, $\:{X}_{2}$ = Stimulus Complexity and $\:{X}_{3}$ = Stimulus Orientation.

Since we had no specific hypothesis concerning the influence of knob resistance on error commission, we ran a third model with the same predictors as the first model for the analysis of error commission.

#### Free choice blocks

In the free-choice blocks, offloading rate as our main dependent variable was analyzed in a logistic mixed model including knob resistance, stimulus complexity and working stimulus orientation. Due to the fact that we only had hypotheses for main effects of each predictor, we compared a model including all interactions to a main effects model to see if possible interactions significantly contributed to the model. This procedure is guided by work of Rimpler et al. (2025). This model comparison revealed that the interactions did not significantly improve the model fit, $\:{{\upchi\:}}^{2}$ (4) = 3.84, *p* =.427. This was also indicated by the AIC ($\:{Model}_{interaction}=4669.6,\:{Model}_{main\:effects}=4665.4$), which was smaller for the model including only main effects. The final model hereafter consisted of only main effects and is formalized as:

3 $$\begin{array}{c}In\;\lbrack P(Offloading)/1-P(Offloading)\rbrack\\=\beta_0+\beta_1X_1+\beta_2X_2+\beta_3X_3\end{array}$$

where $\:{X}_{1}$ = Knob Resistance, $\:{X}_{2}$ = Stimulus Complexity and $\:{X}_{3}$ = Stimulus Orientation.

To analyze response times, we included the selected strategy, stimulus complexity, knob resistance and stimulus orientation as predictors in a linear mixed model. Since we saw the possibility that stimulus complexity, stimulus orientation and knob resistance may influence mentally solved trials differently than offloaded trials, we included their two-way interactions with the selected strategy as well as the three-way interaction of stimulus complexity, stimulus orientation and selected strategy which would point to a difference in the complexity effect between both strategies. We then also compared this model to a model including all possible interactions. The model comparison between our proposed model and the full model showed that our model was better able to explain the data, $\:{{\upchi\:}}^{2}$ (7) = 3.14, *p* =.872. This was also indicated by the AIC ($\:{Model}_{proposed}=17249,\:{Model}_{full}=17260$). As our proposed model was superior to the full model we used the proposed model:

4 $$\begin{array}{c}Response\;Time=\beta_0+\beta_1X_1+\beta_2X_2+\beta_3X_3+\beta_4X_4\\+\beta_{12}X_1X_2+\beta_{13}X_1X_3++\beta_{14}X_1X_4+\beta_{124}X_1X_2X_4\end{array}$$

where $\:{X}_{1}$ = Selected Strategy, $\:{X}_{2}$ = Stimulus Complexity, $\:{X}_{3}$ = Knob Resistance and $\:{X}_{4}$ = Stimulus Orientation.

Error rates were analyzed in another logistic mixed model including the selected strategy, stimulus complexity and stimulus orientation as predictors. Here we included all possible interactions.

5 $$\begin{array}{c}In\lbrack P(Error)/1-P(Error)\rbrack=\beta_0+\beta_1X_1+\beta_2X_2+\beta_3X_3+\\\beta_{12}X_1X_2+\beta_{13}X_1X_3++\beta_{23}X_2X_3+\beta_{123}X_1X_2X_3\end{array}$$

where $\:{X}_{1}$ = Selected Strategy, $\:{X}_{2}$ = Stimulus Complexity and $\:{X}_{3}$ = Stimulus Orientation.

All data analyses were conducted in R (Version 4.3.3, R Core Team, 2021). We used the conventional significance level of α = 0.05. Logistic and linear mixed models were conducted using the *lme4* package (version 1.1–35.1, Bates et al., 2015). Planned pairwise comparisons were estimated using the package *emmeans* (version 1.11.0, Lenth et al., 2025).

## Results

### Forced-Choice blocks

#### Response times and error rates

In the forced-choice blocks response times for offloaded blocks were slightly higher than for the mentally solved block (*β* = 0.27, *t* = 3.45, *p* <.001). High-complexity stimuli (*β* = 0.60, *t* = 12.02, *p* <.001) and an increased working stimulus orientation (*β* = 0.36, *t* = 8.36, *p* <.001) increased response times. The two-way interaction of strategy and stimulus complexity also reached significance (*β* = −0.25, *t* = −2.23, *p* =.026), indicating that the difference in response times between high and low complexity stimuli was greater in the mental forced-choice block compared to the manual forced-choice blocks. The three-way interaction of strategy, stimulus complexity and working stimulus orientation did not reach significance (*β* = −0.02, *t* < 1, *p* =.889). Within the manual forced choice blocks, response times in the high knob resistance block are higher than in the low knob resistance block (*β* = 0.90, *t* = 12.79, *p* <.001). Both stimulus complexity (*β* = 0.52, *t* = 7.41, *p* <.001) and working stimulus orientation (*β* = 0.21, *t* = 3.38, *p* <.001) remained significant in this model. A two-way interaction between knob resistance and working stimulus orientation reached significance (*β* = 0.25, *t* = 2.94, *p* =.003), indicating that the influence of knob resistance was higher for lower stimulus misorientations. Tables with results of all conducted analyses can be found in Online Resource 1.

The error rate was significantly larger in the mental forced-choice block as compared to the manual forced-choice blocks (*β* = −1.62, *z* = −10.82, *p* <.001). While stimulus complexity (*β* = −0.03, *z* < 1, *p* =.804) and working stimulus orientation (*β* = 0.07, *z* < 1, *p* =.512) did not influence error rate, the two-way interaction of strategy and stimulus orientation reached significance, (*β* = −0.62, *z* = −3.40, *p* <.001), indicating that increasing stimulus orientation selectively increased error rate in the mental forced choice block. Neither the two-way interaction of stimulus complexity and working stimulus orientation (*β* = −0.21, *z* = −1.37, *p* =.170) nor the three-way interaction of all predictors reached significance (*β* = 0.21, *z* < 1, *p* =.427).

### Free-Choice blocks

#### Response times and error rates

Within the free-choice blocks offloading a trial took participants significantly longer to do than solving a task mentally ($\:{M}_{offload}$ = 5350 ms, $\:{M}_{mental}$ = 3610 ms), *β* = 1.23, *t* = 11.23, *p* <.001. Furthermore, high stimulus complexity increased response times significantly (*β* = 0.43, *t* = 8.63, *p* <.001). We also observed an increase in response times the further away the working stimulus was rotated (*β* = 0.21, *t* = 6.78, *p* <.001). While high rotation knob resistance led to higher response times (*β* = 0.36, *t* = 7.25, *p* <.001), it did so more strongly for offloaded trials compared to mentally solved trials as indicated by the significant interaction of strategy and rotation knob resistance (*β* = 0.76, *t* = 5.76, *p* <.001). Figures of response times and error rates of Experiment 1 for both forced- and free-choice data can be found in Online Resource 2.

Error rates were smaller, when trials were offloaded (7.6%) than when they were not (16.1%, *β* = −1.10, *z* = −6.54, *p* <.001). Both stimulus complexity (*β* = 0.38, *z* = 6.36 *p* <.001) and increasing working stimulus orientation led to higher error rates (*β* = −0.20, *z* = −2.14, *p* =.032). The model furthermore revealed that there is an interaction between the selected strategy and stimulus orientation (*β* = −0.56, *z* = −2.81, *p* =.005), that is with increasing stimulus orientation error rates increase more strongly for mentally completed trials compared to offloaded trials.

#### Strategy selection

**Fig. 4:**
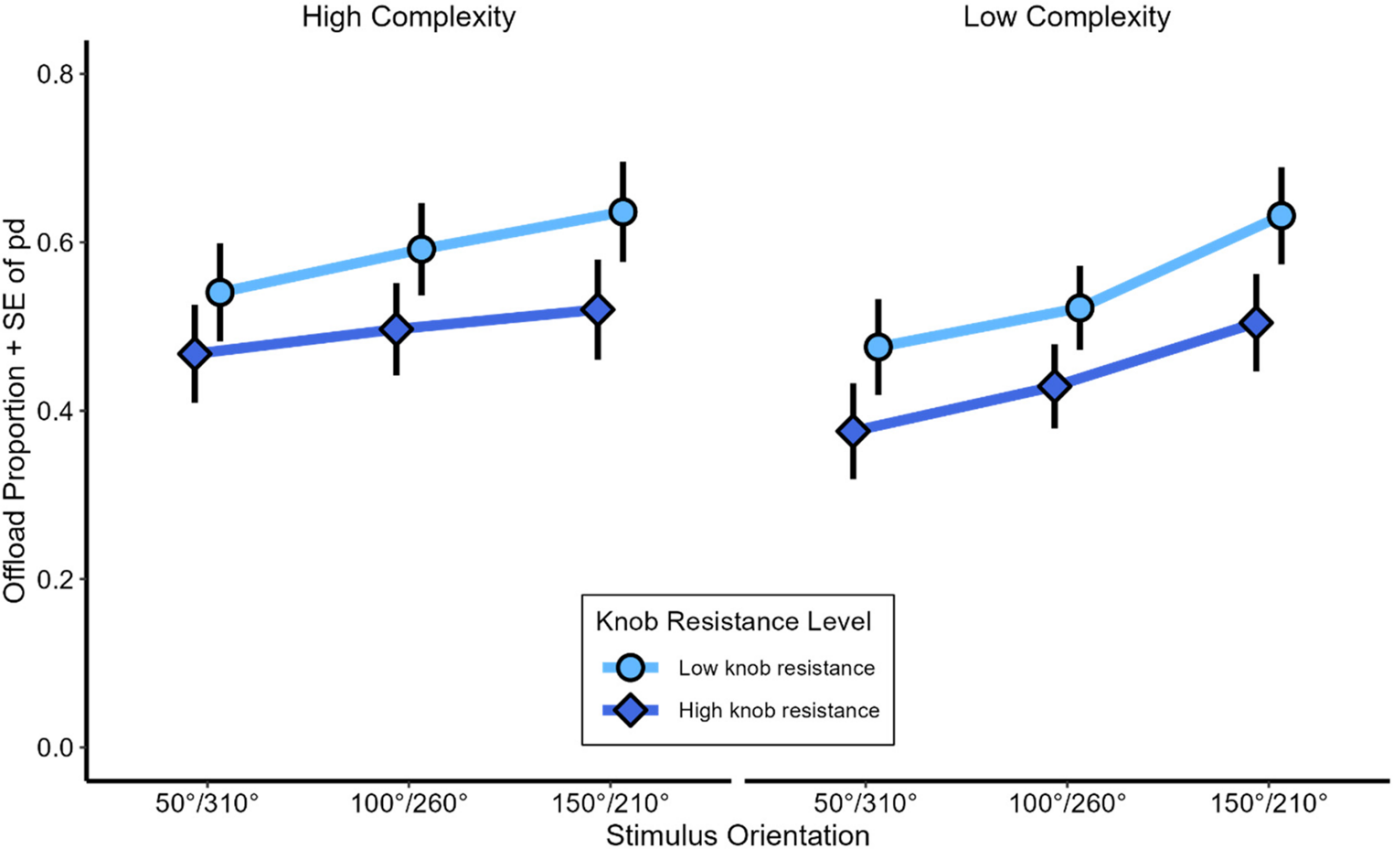
Offloading Rate in Experiment 1. Mean Offloading Rate in free-choice trials as a function of stimulus orientation, the rotation knobs’ resistance and stimulus complexity. Error bars depict standard errors of paired differences for each level of stimulus orientation separately (Pfister & Janczyk, 2013)

Choice frequencies did not indicate an overall preference for either strategy ($\:{M}_{offload}$ = 52.4%, $\:{SD}_{offload}$ = 29.3%). In the low knob resistance condition, participants offloaded 57.1% of all trials, while in the high knob resistance condition individuals offloaded 47.8% of all trials (*β* = −0.63, *z* = −7.65, *p* <.001; see Fig. 4). The main effect of stimulus complexity was also significant (*β* = 0.27, *z* = 3.45, *p* <.001), with high stimulus complexity leading to a higher offloading rate (54.4%), compared to low stimulus complexity (50.6%). Furthermore, the further away the working stimulus orientation was from the 0°-orientation, the more participants offloaded (*β* = 0.35, *z* = 7.01, *p* <.001), with every additional 50 ° of stimulus orientation increasing the odds of offloading by about 36%. These results indicate that the influence of increasing knob resistance (*β* = 0.63) was considerably larger than that of stimulus complexity (*β* = 0.27). Since the two levels of knob resistance correspond to a difference of 0.6 Newton meters (Nm), changing the level of stimulus complexity from low to high has a comparable effect on the log odds of offloading as increasing the resistance of the rotation knob by about 0.26 Nm. Finally, we did not find any indication that the counterbalanced variables (knob position mapping, hand use, answer knob mapping) influenced response times, error rates or strategy selection in a systematic way.

#### Post-error strategy choice

In an exploratory analysis we conducted another logistic mixed model trying to predict whether participants switched their strategy compared to the previous trial (yes or no) through two additional factors, namely whether the task was offloaded in the previous trial (yes or no) and whether it had been solved correctly (yes or no). These two factors as well as their two-way interaction were included in the model described in the regression Equation (3).

This analysis revealed that greater stimulus misorientation led to (*β* = 0.10, *z* = 2.29, *p* =.022) more strategy switches in the subsequent trial. Neither knob resistance (*β* < 0.01, *z* < 1, *p* =.953) nor stimulus complexity (*β* = 0.06, *z* = 1.74, *p* =.082) significantly predicted participants’ switch behavior. However, switching the strategy when trial $\:{\text{N}}_{-1}$ had been solved correctly was at 25.5%. This rate increased to 32.1% when an error had been committed in trial $\:{\text{N}}_{-1}$, which meant a significant difference in switch rate (*β* = 0.88, *z* = 7.06, *p* <.001). Furthermore, the selected strategy of the previous trial also affected switching the strategy (*β* = 0.73, *z* = 8.78, *p* <.001), that is people generally repeated the strategy they had selected in the previous trial. Crucially, the two-way interaction of both predictors reached significance (*β* = −1.00, *z* = −4.08, *p* <.001), indicating that the decision to switch strategies after error commission depends on the strategy of the previous trial.

**Fig. 5:**
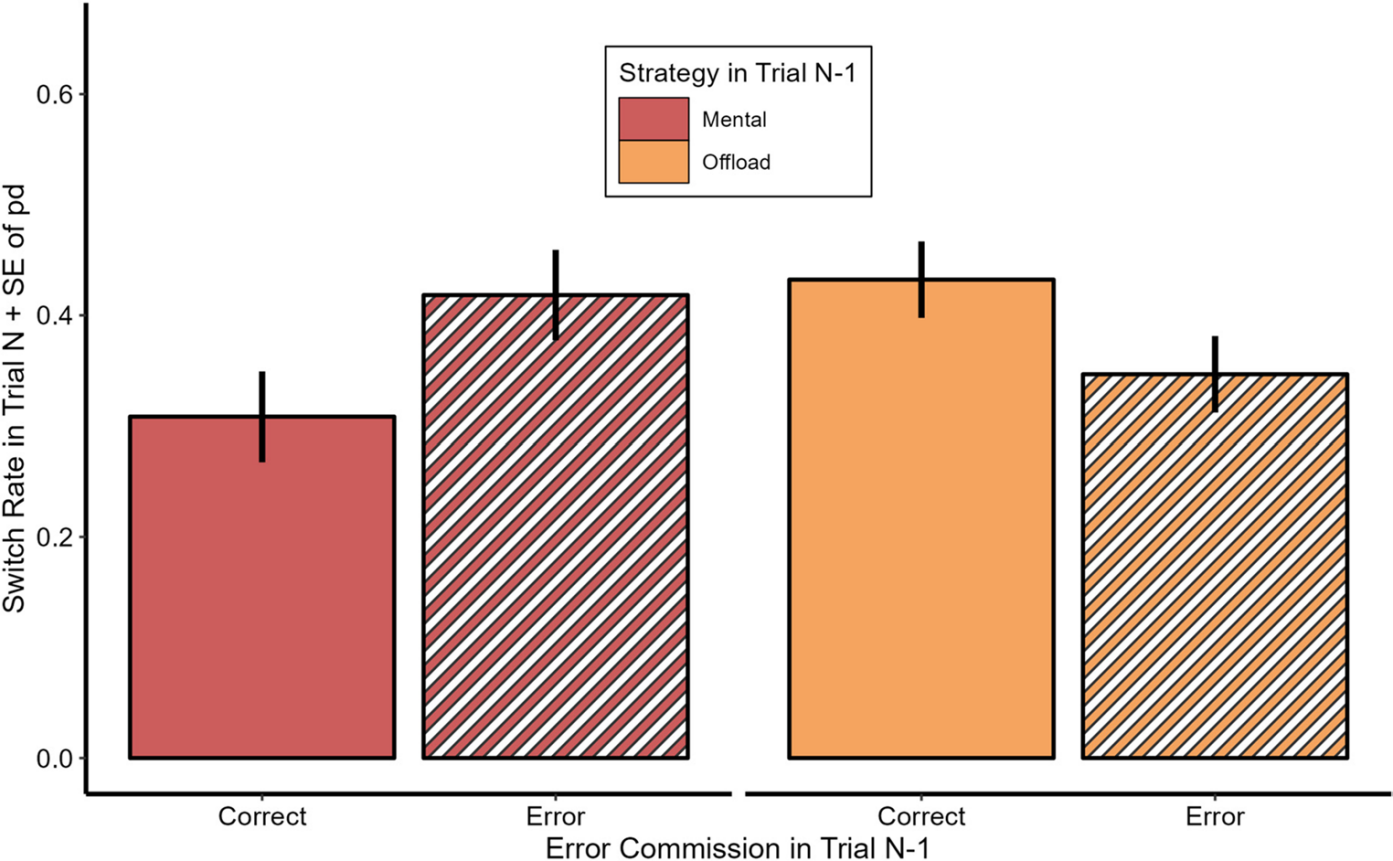
Switch Rates in Experiment 1. Mean Strategy Switch Rate in Trial N as a function of Strategy and Error Commission in Trial N-1. Error bars depict standard errors of paired differences for the mental and offload strategy separately (Pfister & Janczyk, 2013)

To better understand this relationship we conducted pairwise comparisons, which revealed that participants were likely to switch strategies after committing a mental error (*β* = 0.82, *z* = 6.65, *p* >.001) but not after errors when offloading the task (*β* = 0.11, *z* < 1, *p* =.607). Results are visualized in Fig. 5.

## Discussion

Experiment 1 replicated several results from the literature. First, performance in mental task completion declined with increasing object misorientation indicating mental rotation. Second, increasing object complexity decreased performance in forced mental rotation blocks suggesting that more complex objects require more mental effort to be processed. Moreover, the fact that stimulus complexity led to a greater increase in response times for mentally solved trials than for offloaded trials, indicates that stimulus complexity increases mental effort more strongly than physical effort. Similarly, error rates also show a significant interaction of strategy with stimulus orientation, suggesting that also stimulus orientation increased mental effort more strongly than physical effort.

Regarding free strategy choice, participants preferred offloading when physical effort in terms of knob resistance was low rather than high. Thus, as expected participants took physical effort of offloading into account. Yet, the manipulation of physical effort, so as in previous studies, came with increased manipulation time, as participants took longer to rotate a knob with high as compared to low resistance. This leaves some ambiguity whether it is physical effort alone or time that determines offloading choice. A point we will address in Experiment 2. Moreover, participants offloaded more with high rather than low stimulus complexity, and with larger rather than with smaller stimulus misorientations. These observations suggest that stimulus complexity as well as stimulus misorientation increase the effort of mental rotation more than the effort of manual rotation.

Furthermore, participants switched more often to offloading after having committed an error in mental task completion as compared to correct mental task completion. Yet, they did not switch to mental task completion more often after having committed an error while offloading as compared to error-free offloading. If anything, they tended to stick to offloading after having committed an error while offloading. So it does not seem the case that committing an error with a certain means of task completion (mental or manual) prompts people moving to the respectively other means, so as reported for voluntary task switching (Spitzer et al., 2022). Rather as error rate was overall lower with offloading, any error seems to shift participants moving toward the ‘safer harbor’, namely offloading. Finally, the experiment also replicated the finding that participants tend to repeat the strategy they used before, often termed perseveration effect (Scarampi & Gilbert, 2020; Schillemans et al., 2010; Weis & Kunde, 2024).

The discussed aspects were decisive for the main objectives of Experiment 2, which were to further reduce the confound of time and physical effort as well as establish a working manipulation of mental effort. In addition, minor changes in the experimental design were made to increase decision uncertainty before any given trial.

## Experiment 2

We sought to replicate the findings of Experiment 1, while making some minor but crucial changes to the operationalization of mental and physical effort, so as to eliminate the confound of physical effort and time as well as establish a clean and sound manipulation of mental effort. To address the confound of physical effort with time, offloading the task now required the rotation knob to be rotated in either direction for a specified period of time, independently of the working stimulus orientation. After the specified time, this resulted in the working stimulus to discretely flip into the canonical position. That way, no matter how far the working stimulus is rotated in the beginning of a trial, the rotation knob always had to be turned for the same amount of time to achieve offloading a trial. Therefore, the only difference between the two physical effort conditions is the resistance of the rotation knob. Moreover, the ‘flip’ duration of the object was the same irrespective of the initial object misalignment. Arguably, object misalignment would thus no more affect manual rotation but only mental rotation. This in turn also simplifies the operationalization of mental effort, because the amount of required physical effort in a specific trial is no longer associated with the working stimulus orientation, which can therefore now serve as a core manipulation of mental effort. The variable of stimulus complexity thus became superfluous and was removed from the experiment design. We chose to use stimulus orientation over stimulus complexity as a manipulation of mental effort because we were unable to find evidence as conclusive as we would have wished to indicate that stimulus complexity solely increases *mental* effort. To deduce this, we had originally hoped to observe an interaction of stimulus complexity and stimulus orientation specifically for mentally solved trials, which would suggest a slower mental rotation process for high compared to low complexity stimuli. Moreover, we felt that existing literature provides stronger evidence for the effect of stimulus orientation on mental effort than for stimulus complexity (e.g. Jansen et al., 2012; Moè & Pazzaglia, 2010).

## Methods

### Participants

We conducted an a priori power analysis using G*Power (Faul et al., 2007) based on the effect of physical effort on strategy selection in Experiment 1, which yielded large effect sizes. Because of the stated confound of time and physical effort, we assumed the effect size of physical effort alone to be smaller. Therefore, in our power analysis we aimed at detecting a medium-sized effect ($\:{\eta}_{p}^{2}=\:0.05$) by means of linear-mixed models. Our power analysis yielded a minimum required sample size of *N* = 48 participants to detect this medium-sized effect with a power of 1-β = 0.95 and α = 0.05. We therefore collected a total of 48 analyzable data sets via an online participant pool management platform of the University of Würzburg. The study took about 30 min. All participants gave their written consent before participation and received financial compensation of 6€. We excluded any participants, for which technical failures of our apparatus (motion tracker) arose in collecting the data (*n* = 2, 3.3%), as well as those who did not meet our inclusion criteria (*n* = 10, 16.6%). Our final sample consisted of 48 participants (33 females, $\:{M}_{age}$ = 26 years, $\:{range}_{age}$ = 20–54 years).

### Procedure and task

To offload the task in Experiment 2 it was necessary to rotate the knob for 750ms in either direction. The result of that would be that as soon as the 750ms had passed the working stimulus discretely flipped into the canonical orientation, so that it could directly be compared to the base stimulus. In the case that one fluid rotation movement did not last 750ms, a second rotation movement would have to be executed to finish rotating the knob for 750ms.

### Stimuli

The variable of stimulus complexity was removed from the experiment design. Due to that fact, now only one level of stimulus complexity was needed, and we relied on high-complexity stimuli alone.

### Design and data analysis

As we were interested in strategy choice, only free-choice blocks were implemented. In this experiment we also measured the duration for which participants turned the knob, if they used it. The data was analyzed in the same way as the free choice trials were in the first experiment, with only the predictor of stimulus complexity removed from all analyses.

Concerning strategy selection we again compared our model 3 (without stimulus complexity) to a model additionally including the two-way interaction of stimulus orientation and knob resistance. This comparison revealed that the interaction did not significantly improve the model fit $\:{{\upchi\:}}^{2}$ (1) = 0.63, *p* =.427. This is also indicated by the AIC ($\:{Model}_{interaction}=4954.0,\:{Model}_{main\:effects}=4952.7$), which is slightly smaller for the model including only main effects of both predictors.

## Results

### Response times and error rates

Response times revealed that offloading a trial took participants significantly longer than solving a task mentally ($\:{M}_{offload}$ = 7670 ms, $\:{M}_{mental}$ = 4860 ms), *β* = 1.75, *t* = 19.03, *p* <.001. Furthermore, the more stimuli were misaligned, the longer it took people to solve the task, (*β* = 0.26, *t* = 8.12, *p* <.001). While high rotation knob resistance led to higher response times (*β* = 0.30, *t* = 5.77, *p* <.001), the two-way interaction of strategy and knob resistance (*β* = 0.96, *t* = 7.86, *p* <.001) indicates that high rotation knob resistance influence response times more strongly in offloaded trials compared with mentally solved trials. Figures of response times and error rates of Experiment 2 can be found in Online Resource 3. In order to give a better understanding of the participants’ behavior during offloaded trials, we additionally report descriptive time data of offloaded trials. On average 4.10 s (SE = 0.28) of a trial were spent between stimulus onset and starting to rotate the rotation knob. After an on average additional 1.59 s (SE = 0.12) participants stopped manipulating the knob and started their movement towards the answer knob, which registered a response after an additional 1.99 s (SE = 0.12).

Error rates revealed, that participants committed more errors when trials were solved mentally, (Δ = 9.8%), *β* = −1.49, *z* = −9.41, *p* <.001. Working stimulus orientation had an influence on errors as expected, that is increased working stimulus orientation led to higher error rates (*β* = 0.19, *z* = 3.38, *p* <.001). The two-way interaction of both predictors also reached significance (*β* = −0.45, *z* = −2.46, *p* =.014), indicating that stimulus orientation influenced the error rate for mentally solved trials more strongly than for offloaded trials.

### Strategy selection

**Fig. 6:**
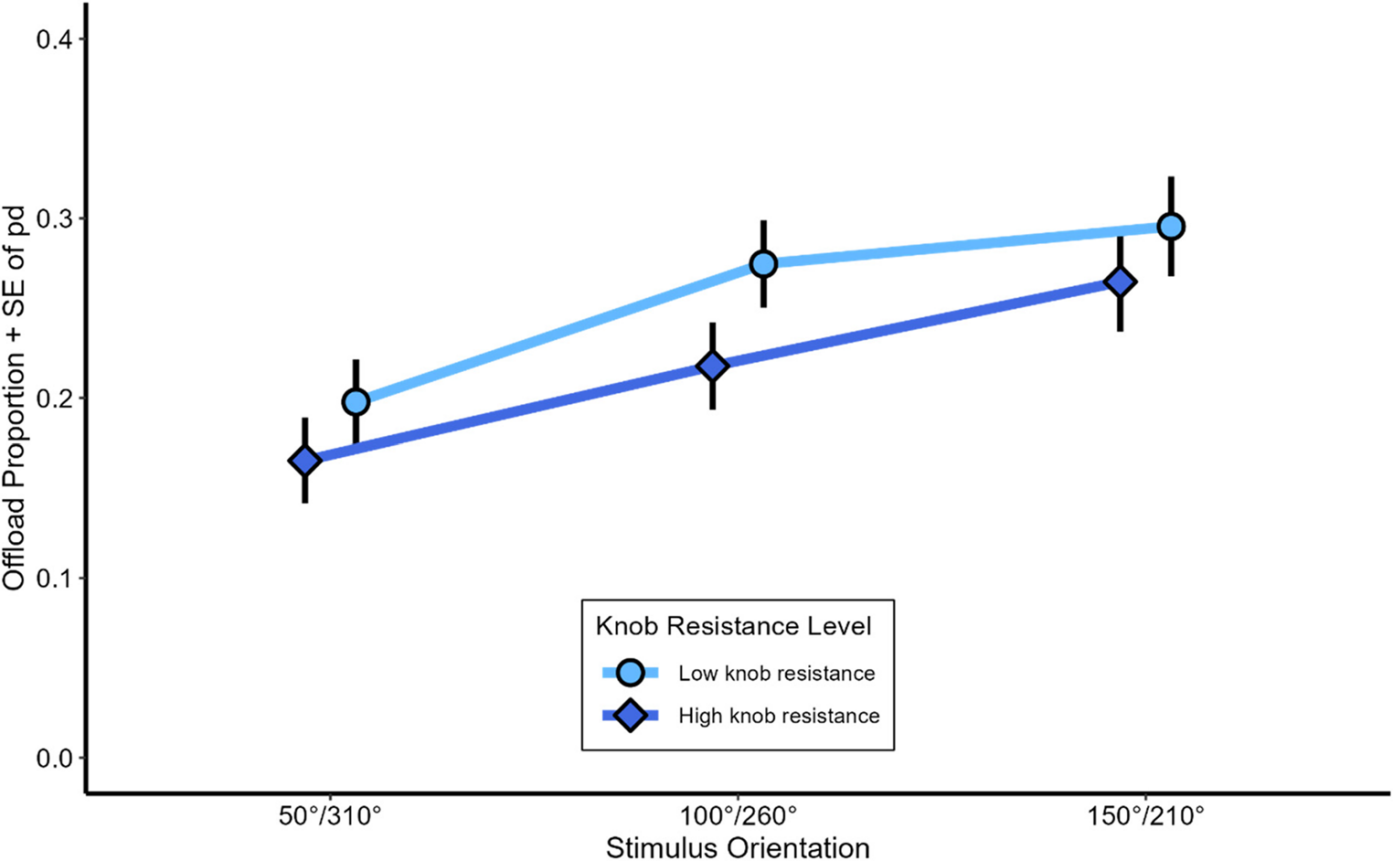
Offload Rate in Experiment 2. Mean Offloading Rate as a function of stimulus orientation and the rotation knobs’ resistance. Error bars depict standard errors of paired differences for each level of stimulus orientation separately (Pfister & Janczyk, 2013)

Choice frequencies indicated that participants preferred using the mental strategy ($\:{M}_{offload}$ = 23.6%, $\:{SD}_{offload}$ = 23.9%). In the low physical effort condition, participants offloaded 26.0% of all trials, while in the high physical effort condition individuals offloaded 21.9% of all trials (*β* = −0.32, *z* = −4.43, *p* <.001; see Fig. 6). Furthermore, the further away the working stimulus orientation was from the 0°-orientation, the more participants used the offloading strategy (*β* = 0.39, *z* = 8.85, *p* <.001).

To check if we successfully removed the confound of physical effort and time we analyzed the duration that participants spent turning the knob. We therefore conducted another linear mixed model in which only offloaded trials were included. As predictors we used the knobs’ resistance and the stimulus orientation. Participants rotated the knob for a longer period of time in the high compared to the low knob resistance condition (∆ = 190ms, *β* = 0.28, *t* = 6.13, *p* <.001). Since we were not sure whether this increased rotation duration in the high knob resistance condition may have also delayed processing the visual effect (the turned working stimulus) and therefore potentially also influence strategy choice, we conducted an additional logistic mixed model to account for this possibility. This model trying to predict strategy selection included the rotation duration as a predictor in addition to knob resistance and working stimulus orientation. This model, controlling for the duration of knob rotation, revealed that all main effects significantly explained strategy selection. While the main effect of knob resistance seemed to be reduced (*β* = −0.16, *z* = −2.00, *p* =.045), the effect of stimulus orientation did not seem to be altered (*β* = 0.39, *z* = 8.82, *p* <.001). The influence of knob rotation duration itself also reached significance (*β* = −0.44, *z* = −3.83, *p* <.001).

### Post-error strategy choice

**Fig. 7:**
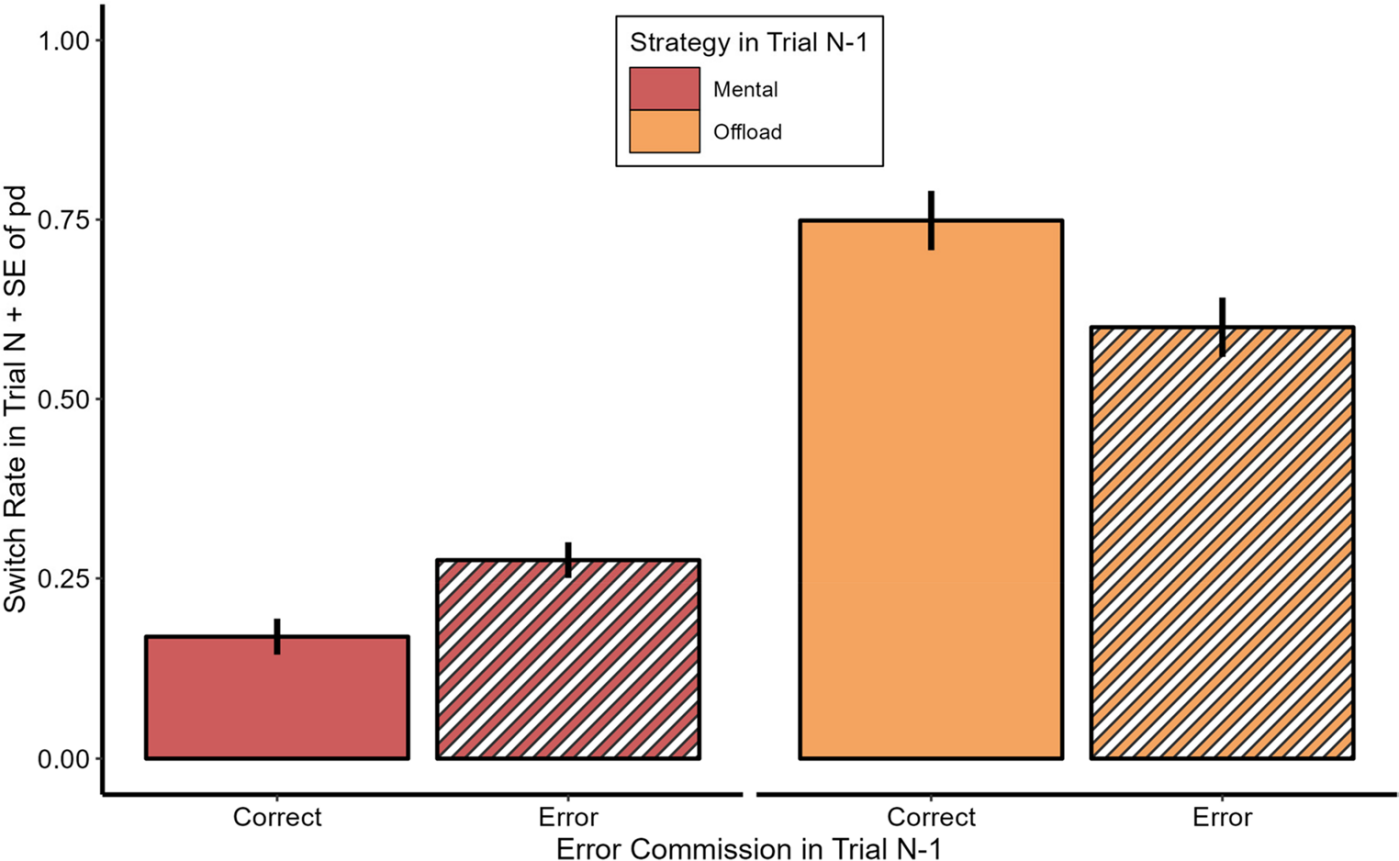
Switch Rates in Experiment 2. Mean Strategy Switch Rate in Trial N as a function of Strategy and Error Commission in Trial N-1. Error bars depict standard errors of paired differences for the mental and manual strategy separately (Pfister & Janczyk, 2013)

The same data pattern as in Experiment 1 emerged concerning switch rates after error commissions as seen in Fig. 7. Individuals showed increased offloading rates for trials after an error had been committed ($\:{M}_{N-1\:correct}$ = 22.5%, $\:{M}_{N-1\:error}$ = 28.3%), *β* = 0.88, *z* = 8.25, *p* >.001. People again had a tendency to use the same strategy as in trial $\:{N}_{-1}$ (*β* = 2.11, *z* = 25.16, *p* <.001). The two-way interaction of both predictors also reached significance here (*β* = −0.90, *z* = −2.75, *p* =.006), again indicating that the decision to switch the strategy after error commissions depended on the previously used strategy.

Post-hoc pairwise comparisons showed that again an error commission when solving the task mentally led to a significant increase in offloading rate in the subsequent trial (*β* = 0.88, *z* = 8.25, *p* <.001). However, even though descriptively an error commission while using the manual strategy led to a decreased switch rate in the subsequent trial, this difference did not reach significance (*β* = 0.02, *z* < 1, *p* =.942). While greater angular mismatch also led to more strategy switches (*β* = 0.23, *z* = 5.44, *p* <.001) knob resistance did not have a significant influence on switch rates (*β* = −0.04, *z* < 1, *p* =.517). Because in both experiments 1 and 2 trials in which participants offloaded and still committed an error were quite rare (7.6% and 3.5% respectively) we deemed it plausible that merely insufficient power prevented the detection of a significant effect. We hence conducted one more logistic mixed model for which the data from both experiments were combined. The qualitative structure of results remained identical to the ones just described. All results concerning this analysis can be found in Online Resource 4.

## Discussion

The main goal of Experiment 2 was to remove the confound of physical effort and time on task. To do so, for each level of physical effort and stimulus orientation the rotation knob had to be rotated for a fixed time period. The results show that the time participants spent rotating the knob still differed significantly, in that in the high compared with the low knob resistance condition participants turned the knob longer. However, when statistically controlling for this difference physical effort still significantly predicted offloading rate. This is evidence for the idea that physical effort in terms of required forces is a factor that is included in peoples’ cost-benefit analyses determining human behavior.

As compared to Experiment 1, overall response latencies increased in Experiment 2, particularly in offloaded trials. While the overall increase is likely due to the use of only highly complex stimuli in Experiment 2, the stronger increase in offloading trials is likely due to the now required minimal knob rotation time which might be higher than the average knob rotation duration in Experiment 1.

Moreover, we replicated our findings from Experiment 1 that incorrect mental strategy trials led participants to switch to offloading in the following trial as compared to correct mental strategy trials. Again, this was not found after errors in offloading trials. It even seems as if participants tended to stay more likely with offloading after erroneous as compared to error-free offloading. However, since this was only a descriptive pattern, we checked if the lack of significance was due to limited power by combining the data of Experiments 1 and 2. This analysis did not show any significant change in switch rate after errors in previous offloading trials.

## General discussion

The present study investigated whether cognitive effort and physical effort are both taken into account when freely choosing between solving a task cognitively or by offloading part of the required cognitive operation to an external device. Indeed, we found that both factors contributed independently. Participants offloaded more when mental rotation required high in comparison to low mental effort. Participants offloaded less when manual rotation required high in comparison to low physical effort. Note that we purposefully never mentioned effort to participants, we controlled for time as a possible confound, and that participants were free to choose whichever strategy they preferred. Even after avoiding these possible confounds, our results suggest that both mental and physical effort are considered when choosing a strategy — even when not explicitly aiming to reduce effort.

As in previous research, it turned out difficult to dissociate required physical effort (in terms of required forces) and duration of effort investment, demonstrating how closely interconnected the two concepts are. Despite careful experimental and statistical control for effort duration, the influence of physical effort on strategy choice persisted. This contradicts the view that neither physical nor cognitive effort but only the time people have to invest in a task determines peoples’ choice behavior as is predicted by the soft constraints hypothesis (Gray et al., 2006; Potts et al., 2018). Our data rather coincides with the view that all three factors cognitive effort, physical effort and time affect decision making (Janczyk at al., 2022). This serves as another indication that great caution seems necessary when designing and interpreting results concerning physical effort. Findings such as that effort is aversive (Oprea, 2020; Vogel et al., 2020) could partly be due to other causes, among them time. This is in line with Pinkston and Libman (2017) arguing that data supporting effort aversion may be due to artifacts in the selected measures. Taken together, on the one hand previous experiments seem to have obtained qualitatively similar findings to ours. On the other hand, these experiments may have still overestimated the effect that physical effort had.

Our results also shed light on the ongoing debate of whether the cognitive system prefers exerting one kind of effort over the other or whether it is impartial to the kind of effort that has to be exerted. When talking about such cognitive impartiality, it may be relevant to agree on the rigour with which the term ‘impartiality’ should be understood. It seems probable that the influence of both kinds of effort is not exactly identical and that identity would be hard to define in the first place. Nevertheless, we question whether partial ideas like the cognitive miser (Kool, 2010) or minimal memory (Ballard, 1997) ideas that suggest a special role of particularly *mental* effort avoidance during problem solving are constructive in their partiality. Instead, our research implies that effort should be viewed in a more holistic manner, spanning both mental and physical domains as well as time. In line with a more holistic view, previous research was not able to find evidence for a bias towards or away from a specific kinds of effort (Potts et al., 2018). There is further support for this view from research suggesting that not the kind of effort itself but the ascribed subjective value for each type of effort determines humans likeliness to selectively invest effort in one domain (Wolff et al., 2024; Inzlicht et al., 2018).

Furthermore, our findings can inform endeavors to discover a common currency that might underly effort-based decisions (e.g., Janczyk et al., 2022; Potts et al., 2018). Though the exact contribution of mental and physical effort is likely task-specific, the present research supplies a data point for the present rotation task, indicating that increasing the force needed to turn the rotation knob by at 1 Newton meter increased offloading rate to the same extent as increasing the angle that needed to be rotated mentally by roughly 35 degrees. As of yet, it is unclear whether our cognitive system engages in such calculations and whether different types of effort are actually combined in a common currency like “inferred effort” (compare Janczyk et al., 2022). However, outcomes like the present one that highlight the human ability to make adaptive choices between mental and physically taxing strategies at least render such calculations plausible. Clearly, more research is needed to support the existence of such calculations as well as their components.

While performance, including errors, has long been isolated as key determinant of offloading choices (e.g., Gray et al., 2006; Gilbert, 2015; Weis & Wiese, 2019), the local influences of errors on offloading choices on the following trial have hitherto mostly been neglected. In the present study, committing an error when using a mental strategy promoted offloading the following trial. On the other hand, committing an error when having offloaded the task did not alter offloading probability in the subsequent trial, with a descriptive tendency to also promote rather than reduce offloading^4^ That errors are important drivers of behavior and underlying choices is well established, which is why we found it surprising that errors during offloading trials had little impact on subsequent strategy choice. And if there was an impact, it was in favor of rather than in opposition to immediately offloading again. Nevertheless, there are plausible theoretical explanations for differences in the way people react to different kinds of errors depending on the utilized strategy. Steinhauser and Kiesel (2011) had shown that there are differences between externally and internally caused errors and that the causal attribution of errors determines post-error adjustments. It could be argued that using the two strategies in this task employ different kinds of resources (Weis & Wiese, 2019; Clark & Chalmers, 1998). While offloading relies on the use of environment-based external resources, mental task completion relies on the use of brain-based internal resources. It seems plausible that the different kinds of errors in our study may therefore be attributed to either external or internal sources as well. A second possible explanation stems from the theory of expected value of control (EVC) which suggests that cognitive control is only implemented if the expected value of the actions consequences is larger than its costs. A study by Matthews et al. (2023) observed increased fatigue and higher experienced cognitive effort after failing at a mental task but not at a physical one. Switching the strategy specifically after a mental error therefore may be another way of avoiding cognitive effort.

Although there is an abundance of literature concerning different consequences of error processing, voluntary task choices after errors have only recently been subject to investigation (e.g. Spitzer et al., 2022; Spitzer et al., 2024; Embrey et al., 2025), which is arguably similar to the voluntary switching of strategies used for the same task (e.g., switching strategies features similar switch costs as switching tasks; Weis & Kunde, 2024). First evidence for adaptive strategy switches comes from Van der Borght et al. (2016), who found that people benefited from using a different strategy to solve math problems after error commissions. Yet, the authors relied on subjective explicit statements of their participants’ mental operation they performed to solve the arithmetic task. Furthermore, strategy switches could not be directly linked to error commissions, that is participants did not report using a different strategy more often after errors than after correct trials. Contrary to this, in our experiment the decision to switch strategies was not only a direct result of having committed an error in trial $\:{N}_{-1}$, but was also directly observed in choice data. To our knowledge post-error offloading seems to be a new and adaptive way for people to react to errors as suggested by the finding that switching ones’ strategy may depend on the previously used strategy. Moreover, researching the underlying psychological processes of post-error offloading would be a meaningful future endeavor. As a limitation, please note that we used trialwise error feedback, which might be the leading cause for error-related strategy switches. However, also note that in task switching, no error feedback is necessary for an error to trigger a task switch (Spitzer et al., 2022).

As a final remark, note that offloading the cognitive task was done to a mechanical device in the present study. In other settings, people might engage in offloading tasks to real or artificial other people (e.g. asking a friend or an AI bot for the solution of a problem). It is an open question whether our findings, especially regarding the continuation of offloading after offloading errors, transfer to such social offloading as well.

## Conclusion

The principle of effort minimization has been widely accepted as one of the key characteristics determining behavior. While the evidence concerning this principle gathered over decades is as conclusive as it seldomly is in psychological research, the ongoingly debated question of whether this is also the case when choosing between different kinds of effort had not been effectively answered. Our study was inspired by pioneering studies investigating the influence of physical effort on task choice. Learning from these studies, we added value by carefully controlling for time as a possible confound with physical effort and by avoiding instructions that install a possibly artificial effort minimization goal in our participants. The present results provide further support for the effort minimization principle, which seems to apply for both mental and physical types of effort. That is, our participants were more inclined to offload the rotation task when physical effort was low vs. high and when cognitive effort was high vs. low. For both experiments, we also explored consistent asymmetric consequences between errors in offloaded versus mentally solved trials: Errors in mentally solved trials served as a trigger to switch to the offloading strategy in the following trial, whereas errors in trials solved with offloading did not trigger switching to the mental strategy.

## Supplementary Information

Below is the link to the electronic supplementary material.

Supplementary file 1 (DOCX 1.49 MB)

## Data Availability

Raw data files and all analyses can be accessed at: https://osf.io/hwb3p/.

## Footnotes

1 Of course, even when manually rotating an object, mental processes similar to those in mental rotation might be involved to some extent. Yet, for the sake of simplicity we denote trials in which the manual offloading option was used as ‘offloaded’ trials.

2 In this pilot study participants chose between mental and manual rotation. The physical effort of manual rotation was manipulated by haying participants pressing a rotation knob either shortly or longer. This manipulation did obviously not meet our goal to vary physical effort independent of time. A description of the study is accessible at OSF (https://osf.io/hwb3p/files/osfstorage).

3 The hand movement trajectory data collected in both experiments is not relevant to the research question addressed here and will therefore not be discussed further.

4 As a note, we would like to acknowledge that error commissions when using the offloading strategy were rather rare in both Experiments 1 and 2 (7.6% and 3.5% respectively), which may lead to a simple power problem when testing for a difference in switch rate. This hypothesis did not receive statistical support in our combined analysis of both experiments’ data.
