# Supplementary material for: Physical effort and task errors influence the choice for cognitive offloading
Source: Psychol Res. 2025 Oct 15;89(5):154. doi: 10.1007/s00426-025-02186-1 (PMC12528289; doi:10.1007/s00426-025-02186-1)
Supplement: Supplementary file 1 — (DOCX 1.49 MB) [file 426_2025_2186_MOESM1_ESM.docx]

**Supplementary Materials**

**Forced Choice Block Analyses**

|  | Experiment 1 | | | |
| --- | --- | --- | --- | --- |
| Fixed Effects | *β* | SE | t | p |
| Intercept | 4.92 | 0.18 | 27.03 | < .001 |
| Strategy | 0.27 | 0.07 | 3.45 | < .001 |
| Stim Complex | 0.60 | 0.05 | 12.02 | < .001 |
| Stim Orient | 0.36 | 0.04 | 8.36 | < .001 |
| Strategy × Stim Complex | -0.25 | 0.11 | -2.23 | .026 |
| Strategy × Stim Orient | -0.10 | 0.10 | -1.01 | .312 |
| Stim Complex × Stim Orient | -0.11 | 0.06 | -1.89 | .059 |
| Strategy × Stim Complex × Stim Orient | -0.02 | 0.14 | -0.14 | .889 |

**Table 1.** Estimate (β), Standard Error (SE), t-value and p-value for each fixed effect (Strategy, Stimulus Complexity and Stimulus Orientation) including their interactions on response times of Model 1 for Experiment 1. × indicates the interaction of two variables.

|  | Experiment 1 | | | |
| --- | --- | --- | --- | --- |
| Fixed Effects | *β* | SE | t | p |
| Intercept | 4.56 | 0.17 | 27.57 | < .001 |
| Knob Resist | 0.90 | 0.07 | 12.79 | < .001 |
| Stim Complex | 0.52 | 0.07 | 7.41 | < .001 |
| Stim Orient | 0.21 | 0.06 | 3.38 | < .001 |
| Knob Resist × Stim Complex | < 0.01 | 0.10 | 0.03 | .978 |
| Knob Resist × Stim Orient | 0.25 | 0.09 | 2.94 | .003 |
| Stim Complex × Stim Orient | -0.12 | 0.09 | -1.45 | .148 |
| Knob Resist × Stim Complex × Stim Orient | 0.01 | 0.12 | 0.04 | .965 |

**Table 2.** Estimate (β), Standard Error (SE), t-value and p-value for each fixed effect (Knob Resistance, Stimulus Complexity and Stimulus Orientation) including their interactions on response times of Model 2 for Experiment 1. × indicates the interaction of two variables.

|  | Experiment 1 | | | |
| --- | --- | --- | --- | --- |
| Fixed Effects | *β* | SE | z | p |
| Intercept | -3.07 | 0.11 | -27.88 | < .001 |
| Strategy | -1.62 | 0.15 | -10.82 | < .001 |
| Stim Complex | -0.03 | 0.12 | 0.25 | .804 |
| Stim Orient | 0.07 | 0.10 | 0.66 | .512 |
| Strategy × Stim Complex | -0.37 | 0.22 | -1.70 | .090 |
| Strategy × Stim Orient | -0.62 | 0.18 | -3.40 | < .001 |
| Stim Complex × Stim Orient | -0.21 | 0.15 | -1.37 | .170 |
| Strategy × Stim Complex × Stim Orient | 0.21 | 0.26 | 0.79 | .427 |

**Table 3.** Estimate (β), Standard Error (SE), z-value and p-value for each fixed effect (Knob Resistance, Stimulus Complexity and Stimulus Orientation) including their interactions on error rates of Model 1 for Experiment 1. × indicates the interaction of two variables.

**Free Choice Block Analyses**

|  | Experiment 1 | | | |  | Experiment 2 | | | |
| --- | --- | --- | --- | --- | --- | --- | --- | --- | --- |
| Fixed Effects | *β* | SE | t | p |  | *β* | SE | t | p |
| Intercept | 4.12 | 0.17 | 23.98 | < .001 |  | 5.21 | 0.26 | 20.34 | < .001 |
| Strategy | 1.23 | 0.11 | 11.23 | < .001 |  | 1.75 | 0.09 | 19.03 | < .001 |
| Knob Resist | 0.36 | 0.05 | 7.25 | < .001 |  | 0.30 | 0.05 | 5.77 | < .001 |
| Stim Complex | 0.43 | 0.05 | 8.63 | < .001 |  |  |  |  |  |
| Stim Orient | 0.21 | 0.03 | 6.78 | < .001 |  | 0.26 | 0.03 | 8.12 | < .001 |
| Strategy × Knob Resist | 0.76 | 0.13 | 5.76 | < .001 |  | 0.96 | 0.12 | 7.86 | < .001 |
| Strategy × Stim Complex | 0.06 | 0.12 | 0.53 | .594 |  |  |  |  |  |
| Strategy × Stim Orient | 0.16 | 0.11 | 1.46 | .144 |  | -0.06 | 0.07 | -0.81 | .420 |
| Strategy × Stim Complex × Stim Orient | -0.32 | 0.15 | -2.11 | .035 |  |  |  |  |  |

**Table 4.** Estimate (β), Standard Error (SE), z-value and p-value for each fixed effect (Strategy, Knob Resistance, Stimulus Complexity and Stimulus Orientation) including specific interactions on response times of Model 4 for each experiment, respectively. In Experiment 2 the fixed effect of stimulus complexity as well as its interactions with other variables are not reported because it was removed from the design. × indicates the interaction of two variables.

|  | Experiment 1 | | | |  | Experiment 2 | | | |
| --- | --- | --- | --- | --- | --- | --- | --- | --- | --- |
| Fixed Effects | *β* | SE | z | p |  | *β* | SE | z | p |
| Intercept | -2.14 | 0.11 | -19.80 | < .001 |  | -2.32 | 0.09 | -25.13 | < .001 |
| Strategy | -1.10 | 0.17 | -6.54 | < .001 |  | -1.49 | 0.15 | -9.41 | < .001 |
| Stim Complex | 0.38 | 0.06 | 6.36 | < .001 |  |  |  |  |  |
| Stim Orient | -0.20 | 0.09 | -2.14 | .032 |  | 0.19 | 0.05 | 3.38 | < .001 |
| Strategy × Stim Complex | -0.05 | 0.24 | -0.19 | .846 |  |  |  |  |  |
| Strategy × Stim Orient | -0.56 | 0.20 | -2.81 | .005 |  | -0.45 | 0.18 | -2.46 | .014 |
| Strategy × Stim Complex × Stim Orient | 0.55 | 0.28 | 1.96 | .050 |  |  |  |  |  |

**Table 5.** Estimate (β), Standard Error (SE), z-value and p-value for each fixed effect (Strategy, Stimulus Complexity and Stimulus Orientation) including their interactions on error rates of Model 5 for each experiment, respectively. In Experiment 2 the fixed effect of stimulus complexity as well as its interactions with other variables are not reported because it was removed from the design. × indicates the interaction of two variables.

|  | Experiment 1 | | | |  | Experiment 2 | | | |
| --- | --- | --- | --- | --- | --- | --- | --- | --- | --- |
| Fixed Effects | *β* | SE | z | p |  | *β* | SE | z | p |
| Intercept | 0.48 | 0.37 | 1.29 | .197 |  | -1.65 | 0.31 | -5.30 | < .001 |
| Knob Resist | -0.63 | 0.08 | -7.65 | < .001 |  | -0.32 | 0.07 | -4.43 | < .001 |
| Stim Complex | 0.27 | 0.08 | 3.45 | < .001 |  |  |  |  |  |
| Stim Orient | 0.35 | 0.05 | 7.01 | < .001 |  | 0.39 | 0.04 | 8.85 | < .001 |

**Table 6.** Estimate (β), Standard Error (SE), z-value and p-value for each fixed effect (Knob Resistance, Stimulus Complexity and Stimulus Orientation) on offloading rates of Model 3 for each experiment, respectively. In Experiment 2 the fixed effect of stimulus complexity is not reported because it was removed from the design.

|  | Experiment 2 | | | |
| --- | --- | --- | --- | --- |
| Fixed Effects | *β* | SE | z | p |
| Intercept | 1.43 | 0.11 | 13.12 | < .001 |
| Knob Resist | 0.28 | 0.05 | 6.13 | < .001 |
| Stim Orient | 0.05 | 0.03 | 1.66 | .098 |

**Table 7.** Estimate (β), Standard Error (SE), t-value and p-value for each fixed effect (Knob Resistance and Stimulus Orientation) on rotation duration for Experiment 2.

|  | Experiment 2 | | | |
| --- | --- | --- | --- | --- |
| Fixed Effects | *β* | SE | t | p |
| Intercept | -1.06 | 0.21 | -4.91 | < .001 |
| Knob Resist | -0.16 | 0.08 | -2.00 | .045 |
| Stim Orient | 0.39 | 0.04 | 8.82 | < .001 |
| Rotation Duration | -0.44 | 0.11 | -3.83 | < .001 |

**Table 8.** Estimate (β), Standard Error (SE), z-value and p-value for each fixed effect (Knob Reistance, Stimulus Orientation and Rotation Duration) on offloading rates for Experiment 2.

**Post Error Strategy Choice**

|  | Experiment 1 | | | |  | Experiment 2 | | | |
| --- | --- | --- | --- | --- | --- | --- | --- | --- | --- |
| Fixed Effects | *β* | SE | z | p |  | *β* | SE | z | p |
| Intercept | -1.89 | 0.26 | -7.22 | < .001 |  | -2.32 | 0.21 | -10.90 | < .001 |
| Knob Resist | < 0.01 | 0.03 | 0.06 | .953 |  | -0.04 | 0.07 | -0.65 | .517 |
| Stim Orient | 0.10 | 0.04 | 2.29 | .022 |  | 0.23 | 0.04 | 5.44 | < .001 |
| Stim Complex | 0.06 | 0.03 | 1.74 | .082 |  |  |  |  |  |
| N-1 Error | 0.88 | 0.12 | 7.06 | < .001 |  | 0.88 | 0.11 | 8.25 | < .001 |
| N-1 Strategy | 0.73 | 0.08 | 8.78 | < .001 |  | 2.11 | 0.08 | 25.16 | < .001 |
| N-1 Error × N-1 Strategy | -1.00 | 0.24 | -4.08 | < .001 |  | -0.90 | 0.33 | -2.75 | .006 |

**Table 9.** Estimate (β), Standard Error (SE), z-value and p-value for each fixed effect (Knob Resistance, Stimulus Complexity, Stimulus Orientation, Error Commission in trial N-1 and Strategy in trial N-1) including one specific interaction on strategy switch rates for each experiment, respectively. In Experiment 2 the fixed effect of stimulus complexity is not reported because it was removed from the design. × indicates the interaction of two variables.


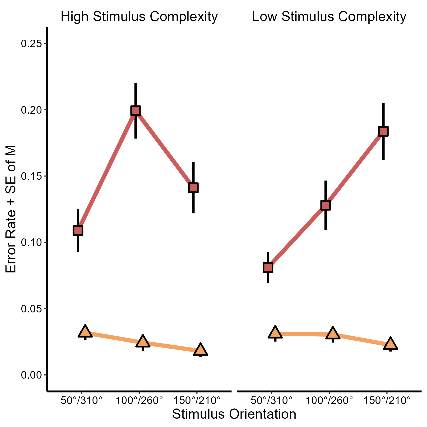

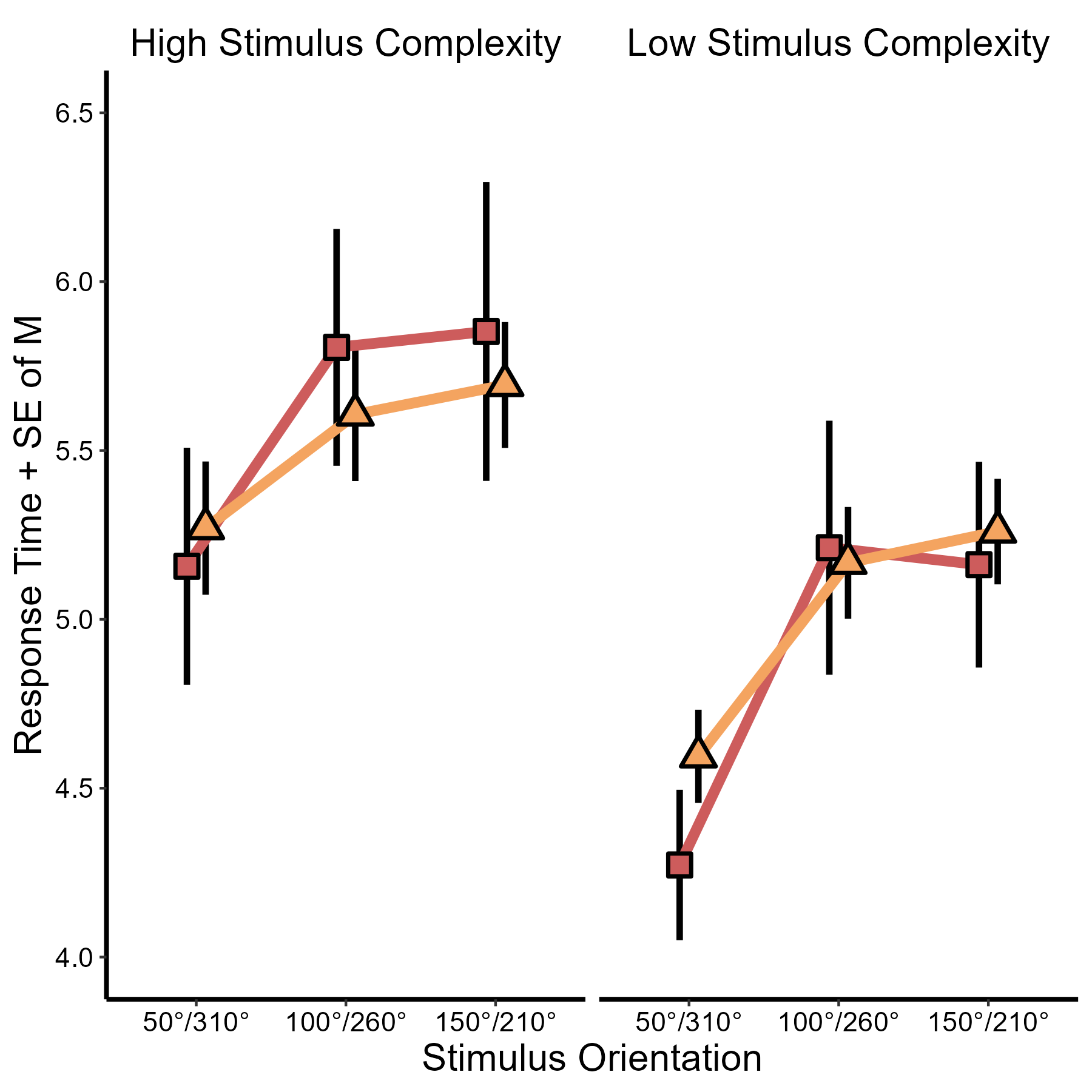

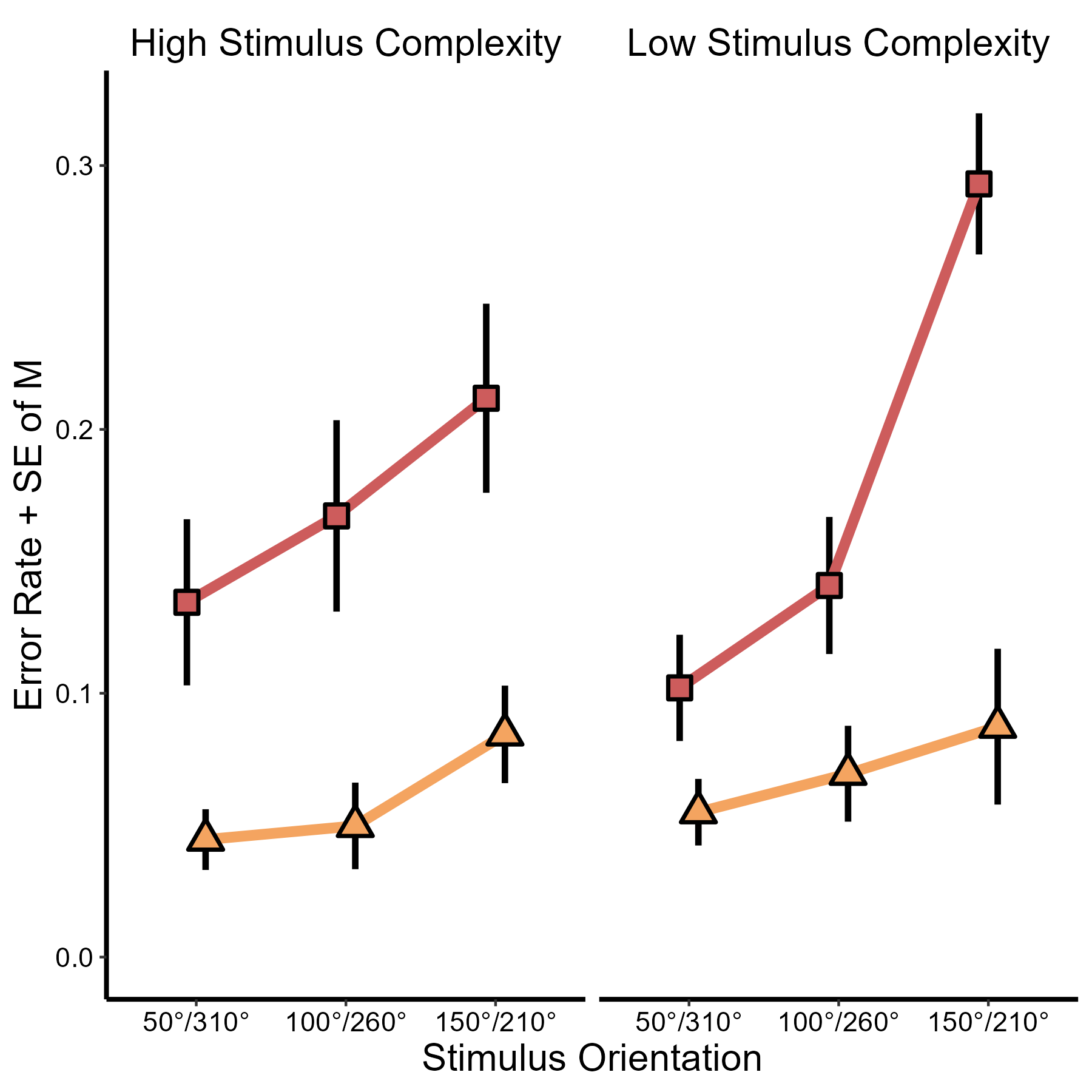


**a**

**b**


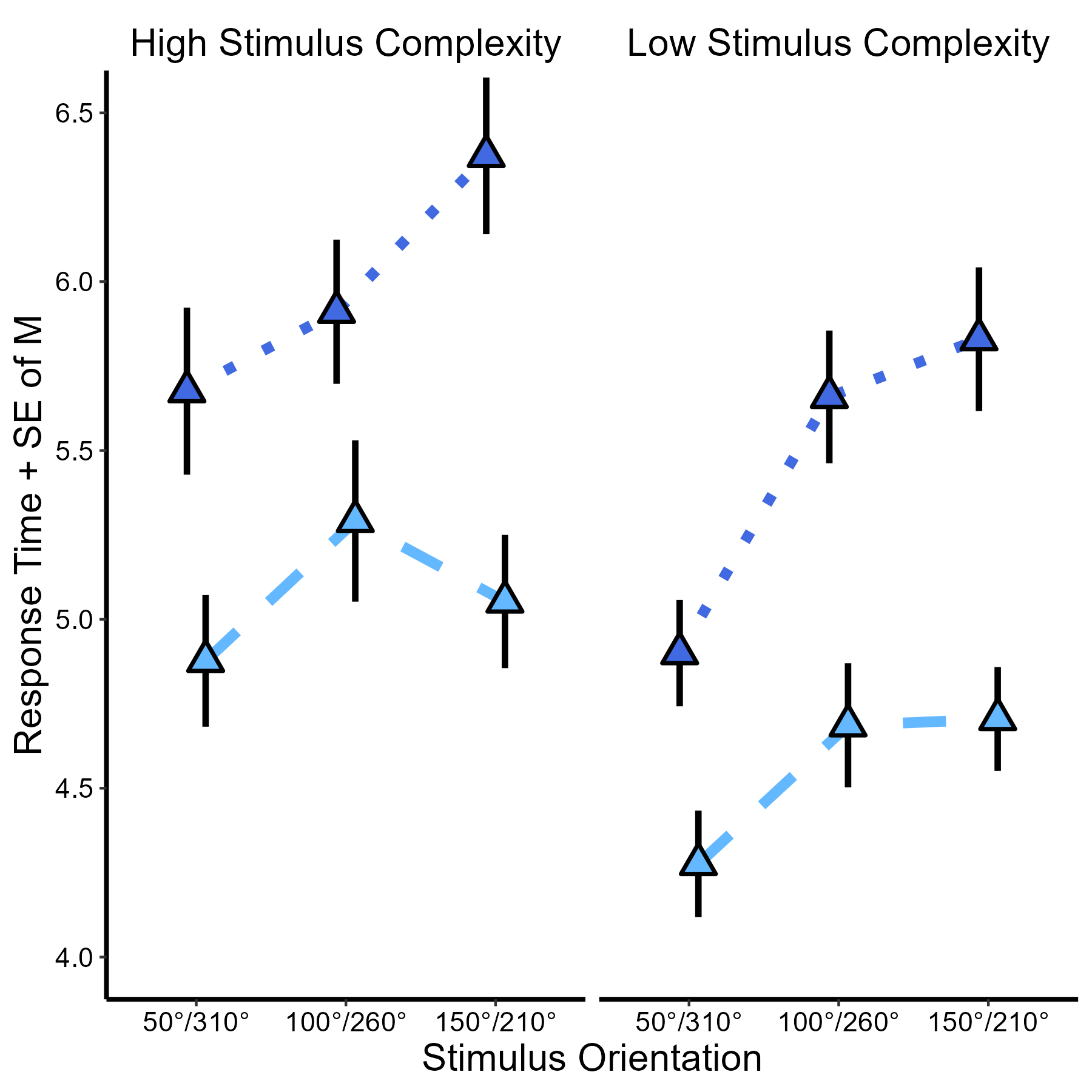

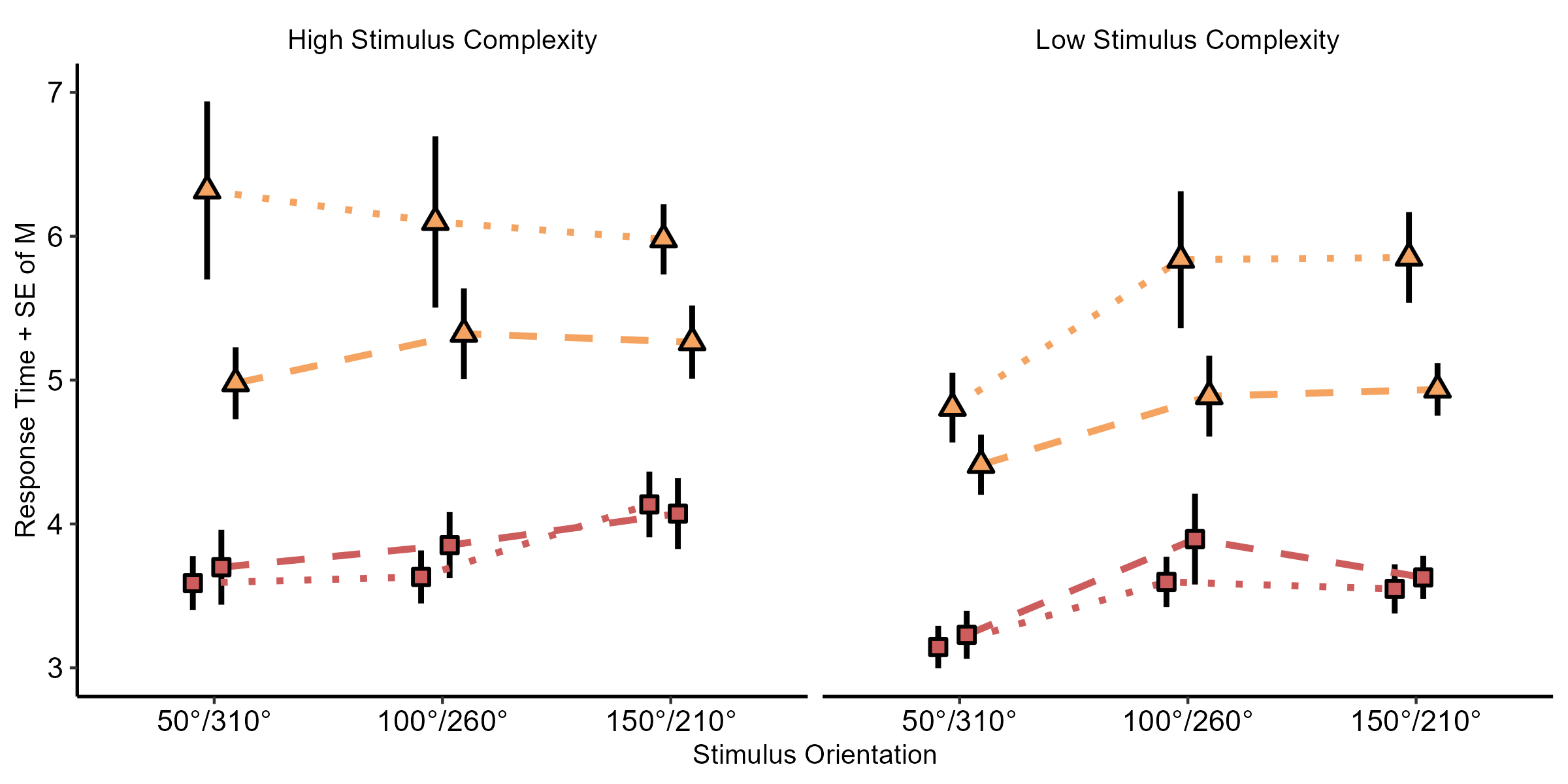


**Fig. 8** Response Times and Error Rates of Experiment 1. Response Times are depicted on the left. Error Rates on the right. All Error Bars depict standard errors of the mean. Note that the y-axis is cut off. Red lines (squares) represent mentally solved trials. Orange lines (triangles) represent offloaded trials. Solid lines represent data averaged across both levels of knob resistance. Dashed lines (also light blue) represent low knob resistance. Dotted lines (also dark blue) represent high knob resistance. a) Forced-Choice Data. b) Free-Choice Data.


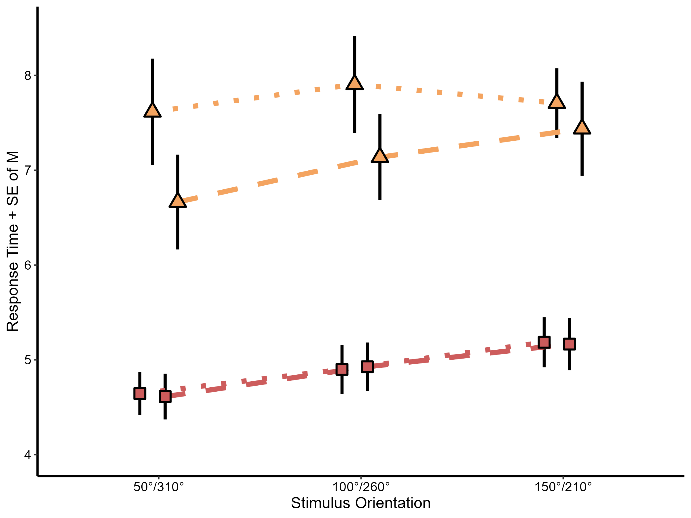

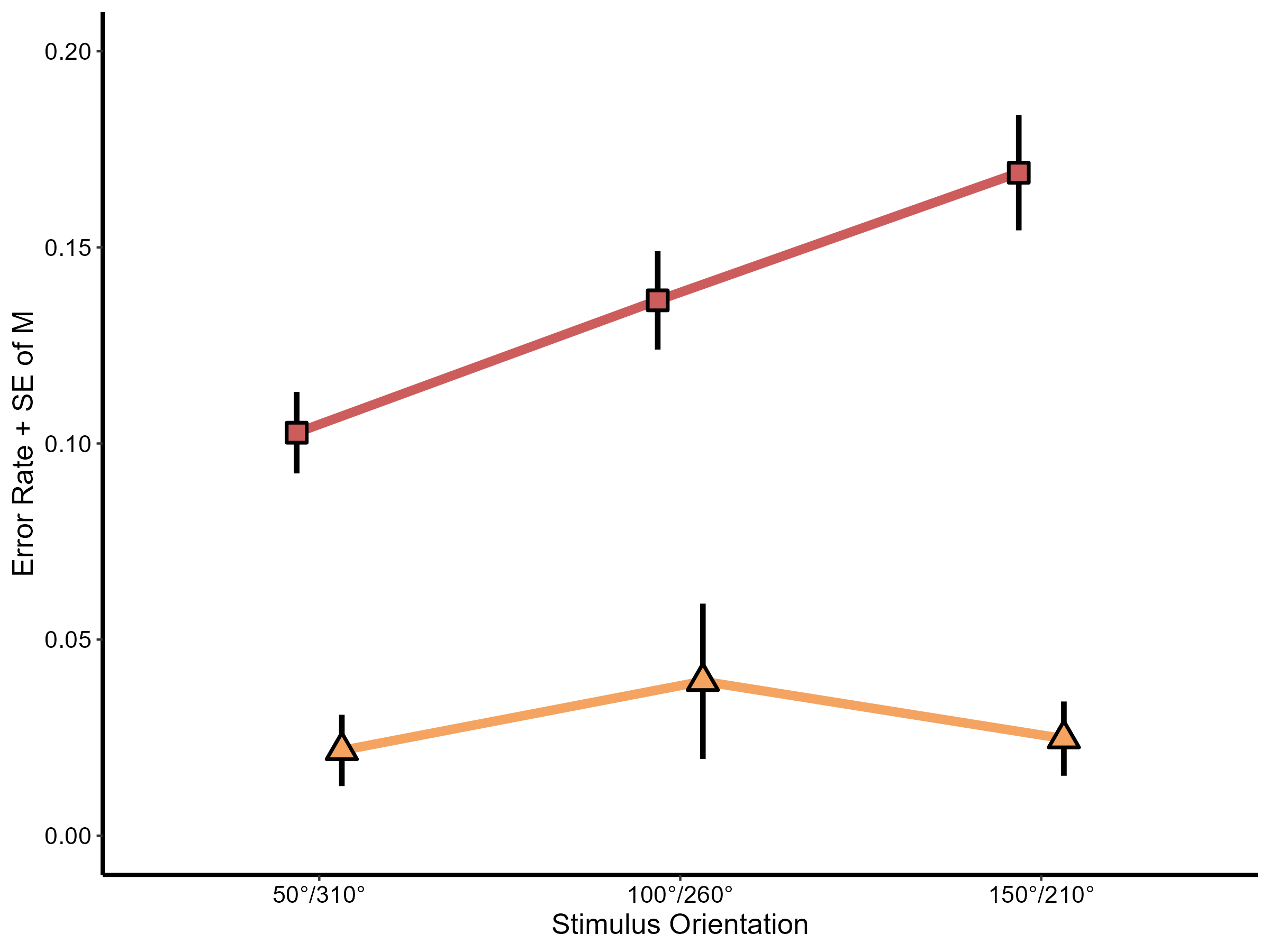


**Fig. 9** Response Times and Error Rates of Experiment 2. Response Times are depicted on the left. Error Rates on the right. All Error Bars depict standard errors of the mean. Note that the y-axis is cut off. Red lines (squares) represent mentally solved trials. Orange lines (triangles) represent offloaded trials. Solid lines represent data averaged across both levels of knob resistance. Dashed lines represent low knob resistance. Dotted lines represent high knob resistance.

**Reanalysis of post-error offloading**

Because in both experiments 1 and 2 trials in which participants offloaded and still committed an error were quite rare (7.6% and 3.5% respectively) we deemed it plausible that merely insufficient power prevented the detection of a significant effect. We hence conducted one more logistic mixed model for which the data from both experiments were combined. In this analysis committing an error again led to higher switch rates (*β* = 0.90, *z* = 11.49, *p* < .001). Furthermore, participants showed an overall tendency to repeat the strategy used in the previous trial (*β* = 1.30, *z* = 23.67, *p* < .001). The two-way interaction of error commission and strategy in the previous trial also reached significance (*β* = 1.00, *z* = 5.51, *p* < .001). Crucially, the contrast of interest between committing an error and correctly solving a trial while offloading did not decrease switch rates significantly (*β* = 0.10, *z* < 1, *p* = .540). Committing an error when mentally solving a trial again led to an increase in switch rate (*β* = 0.90, *z* = 11.49, *p* < .001).
